# Optimization for esterification of saturated palm fatty acid distillate by D-optimal design response surface methodology for biolubricant production

**DOI:** 10.3906/kim-2103-11

**Published:** 2021-06-04

**Authors:** Majd Ahmed JUMAAH, Nadia SALIH, Jumat SALIMON

**Affiliations:** Department of Chemical Sciences, Faculty of Science and Technology, Universiti Kebangsaan Malaysia, Selangor, Malaysia

**Keywords:** Biolubricant, saturated palm fatty acid distillate, polyhydric alcohol, D-optimal design

## Abstract

This work presents a synthesis of palm fatty acid distillate (PFAD)-based esters to produce biolubricant oils through the esterification reaction between saturated palm fatty acid distillate (SFA-PFAD) with different types of high degree polyhydric alcohols such as trimethylolpropane (TMP), di-trimethylolpropane (Di-TMP), pentaerythritol (PE), and di-pentaerythritol (Di-PE) in the presence of sulfuric acid as catalyst. The chemical structures of synthesized SFA PFAD-based esters were characterized and confirmed by using FTIR, NMR (^1^H and ^13^C) spectroscopies and GC-FID chromatography. The FTIR spectra of SFA PFAD-based ester products clearly showed the peaks of C=O and C–O of ester group at 1732–1740 cm^−1^ and at 1239–1162 cm^−1^, respectively. Furthermore, ^1^H NMR spectra confirmed the proton chemical shift (-CH_2_-O-) of the ester group at 3.80–4.01 ppm. The ^13^C NMR spectra confirmed the carbon chemical shifts of ester carbonyl signals at 171.09–174.07 ppm and secondary carbons (CH_2_-C = O) at 40.57–42.44 ppm. The results showed that the optimum conditions for the esterification of SFA-TMP was obtained at acid catalysts of 5%, esterification time and temperature of 6 h and 150 °C, respectively. The results have shown the ester products yields have been significantly increased up to 93% with selectivity of 99% SFA-TMP tri-ester after the optimization process by using D-optimal design. The results for lubrication properties have shown that the SFA PFAD-based esters have low-temperature properties with pour points value in the range of 18–35 °C, flash point (270–310 °C), onset oxidative stability temperature (251–322 °C) and viscosity indices (115–131), respectively. The results showed that the presence of many esters functional groups in the molecule structure of SFA PFAD-based esters provides a positive impact on the lubrication properties. Overall, the results indicated that the SFA PFAD-based esters can be used as biolubricant base oils with pour point depressants.

## 1. Introduction

Lubricants can be found in a variety forms including gases, liquid products (mineral oils, animal and plant oils, derivatives of fatty acids, synthetic oils, and water-based fluids), greases (complex soap greases, greases with pigment, minerals, and polymers) and solid lubricant (graphite and Teflon). In many applications, the liquid form, semi-liquid form, and grease are used to resist friction and wear. However, in extreme service conditions (i.e., very high or low temperatures, vacuum, radiation, extreme contact pressure), biodegradable liquid esters or solid lubricants are selected [1].

Ester is an oxygen-containing organic compound formed through the reaction of an alcohol with an organic acid. Among synthetic esters, polyhydric alcohol esters are of particular interest due to their excellent lubrication properties as low volatility, high flash point, high viscosity index, good thermal stability, and low toxicity. Ester compounds have been used in a range of commercial products such as biodiesel and other alternative diesel fuels derived from plant oils or animal fats [2]. The lubrication properties of esters depend upon the balance between the nonpolar hydrocarbon moiety and the polar ester groups. The hydrocarbon chain length is reflected in the increase of viscosity, viscosity index, and flash point, while the pour point and oxidative stability temperature are affected primarily by the existence of saturated and unsaturated fatty acids [3]. The polarity of an ester group plays an important and significant effect on the material vapor pressure, lubricity, and solubility [4]. In comparison to mineral oils, many esters have a series of technical advantages. These advantages include a naturally high viscosity index, enhancement of low-temperature properties, volatility, oxidative stability, hydrolytic stabilities, antifriction capabilities, and antiwear [5]. Bulky moiety ester compounds have been synthesized and derived from plant oils or animal fats-based raw materials.

Palm fatty acid distillate (PFAD) is a by-product of refining crude palm oil (CPO). PFAD is a light brown semi-solid at room temperature melting to a brown liquid on heating. PFAD comprises mainly mixtures of free fatty acids (FFA) (>80%). These large amount of free fatty acids (FFA) in PFAD have been recovered from the deodorization process. Oleochemical industries use PFAD as starting material to produce an intermediate product to make plastic, animal feed, and as the raw material for medium-grade cleaners. Some researchers have pointed out toward the promise of using FFA extracted from PFAD to produce biodiesel [6–9].

PFAD comprise mainly with saturated palmitic acid and unsaturated oleic acid (about 84% total) as the major fatty acids composition. The remaining components are partial glycerol and unsaponifiable matters, vitamin E, sterols, squalenes, and volatile substances [10]. Surveys on the characteristics and properties of PFAD from Malaysia refineries have been conducted by Ping and Yusof [11] showed that PFAD consist of more than 80% free fatty acid comprises C_14:0_ (1.2%), C_16:0_ (46.9%), C_16:1_ (0.15%), C_18:0_ (4.3%), C_18:1_ (36.7%), C_18:2_ (9%), C_18:3_ (0.3%), C_20:0_ (0.2%), and others (0.1%). This study seems to be in agreement with the findings by Majd et al. [12] and Baharudin et al. [13], which indicated that palmitic acid (47.1%) was the dominant fatty acid in the Malaysian PFAD, followed by oleic acid (36.6%), and linoleic acid (9.6%). These important fatty acids can be used as low-cost starting materials to produce high-end products such as biolubricants. The remaining composition PFAD consists of 14.4% glycerol, 0.5% vitamin E, 0.8% squalene, 0.4% sterols, and 2.2% others [14].

Biolubricants based on saturated fatty acids often have high oxidative stability and flash point [5,12]. This is due to the properties of saturated fatty acids with high oxidative stability and flash point led to produce biolubricant with good lubrication. Therefore, this study used saturated palm fatty acids distillate (SFA-PFAD) separated from palm fatty acid distillate (PFAD) to produce biolubricants. Several possible types of esters (mono, di-, poly-, and complex esters) might be classified as biodegradable lubricants [15]. However, there is no comprehensive study on polyhydric alcohol-based esters from saturated palm fatty acids. Many researchers have recognized the need to establish the role of polyhydric alcohol-based esters in the field of industrial application biolubricants. The work on the synthesis of saturated palm fatty acid distillate (SFA-PFAD)-based esters was carried out and the aims of this work is to discuss the optimization of the esterification process between saturated palm fatty acid distillate and polyhydric alcohols by using response surface methodology based on the D-optimal approach. The effect of the esterification parameters such as the reaction temperature, reaction time, substrate mole ratio, amount of catalyst to be determined to obtain the highest ester yield. The chemical structures of saturated fatty acid and alcohols used (trimethylolpropane (TMP), di-trimethylolpropane (Di-TMP), pentaerythritol (PE), and di-pentaerythritol (Di-PE)) on the resultant esters lubrication properties were studied.

## 2. Materials and methods

### 2.1. Materials

Palm fatty acid distillate (PFAD) was obtained from a local refinery, Sime Darby Plantation Berhad (647766-V), located at Selangor, Malaysia. Saturated palm fatty acid distillate (SFA-PFAD) represented by palmitic acid, 88.5% were separated from PFAD by low-temperature methanol crystallization method [12]. Polyhydric alcohols, toluene, sulfuric acid (97%), sodium bicarbonate, ethyl acetate, sodium chloride, and anhydrous sodium sulfate were purchased from Sigma Aldrich (Steinheim, Germany). All the chemicals used in this study were either analytical grade or high performance liquid chromatography (HPLC) grade and used directly without further purification.

### 2.2. Instrumentation

The analysis of synthesized ester compound was performed to: i) confirm and determine the optimized ester structure; and ii) determine the composition of ester. In the first step, nuclear magnetic resonance (NMR) and fourier transform infrared spectroscopy (FTIR) were employed, FT-NMR (^1^H and ^13^C NMR) analysis were recorded on JEOL-ECP 400 spectrometer using CDCl_3_ as the solvent. The FTIR spectra were recorded on Perkin Elmer Infrared Spectrophotometer within the range between 500 to 4000 cm^−1^. Gas Chromatography (DB-5HT (30 m × 0.25 mm × 0.25 μm) equipped with a Flame Ionization Detector (GC-FID) was used in the second step. The sample preparation involved the addition of exactly 0.5 mL ester to a vial of 10 mL capacity, after which the mixture was diluted using GC grade ethyl acetate (5 mL). An initial temperature of 100 °C was set for the oven, and then kept constant for 1 min. Then, the temperature was increased to 380 °C by a step increment of 5 °C/min and then kept constant for 25 min. The column temperature was adjusted at 100 °C and increased to 380 °C with a temperature rate increase of 5 °C/min and step increase of about 1 °C. The temperature was then kept constant for 20 min upon reaching 380 °C. Temperatures of 380 and 400 °C were set as the injector and detector temperatures, respectively. The carrier gas for the GC system, helium, was injected at a flow rate of 1 μL of sample. The parameters of GC were carried out according to Nowicki et al. [16]. The peaks were identified by comparing the retention times to authentic standards.

### 2.3. Esterification of saturated palm fatty acid distillate with high degree polyhydric alcohols

Biolubricant was synthesized from the esterification reaction between saturated palm fatty acids distillate (SFA-PFAD) with various high degree polyhydric alcohols, TMP, Di-TMP, PE, and Di-PE. The amounts of polyhydric alcohols used are calculated according to the mole ratio towards the SFA-PFAD in excess manner as shown in Table 1. For the esterification between SFA-PFAD and TMP, for example was carried out as follow. In a flask with a reflux condenser and three necks, SFA-PFAD (0.037 mol; 104.7 g) was mixed with 1 mole trimethylolpropane (0.037 mol; 5g) at mole ratio of 3.5:1 in Dean-Stark distillation unit. The esterification was carried out at reaction temperature between 110–150 °C in oil bath equipped with stirrer magnetic heater. At required temperature, 5% concentrated H_2_SO_4_ (as a percentage of the weight of SFA-PFAD) was added at specific reaction time. About 20–30 mL of toluene as azeotrope distillation agent was then slowly added to the mixture during the esterification process. After the reaction end at 6 hr, the flask was allowed to cool at room temperature, followed with the removal of toluene by using rotary evaporator at 100 °C. The reaction product was dissolved into 100 mL of ethyl acetate and transferred into a 150 mL separation funnel. About 30 ml of saturated sodium bicarbonate (NaHCO_3_) was added to the separation funnel and shaken for neutralization of the remaining SFA-PFAD and acid catalyst. The funnel separator was left until two layers formed. The aqueous layer at the bottom was removed, leaving the organic layer. The organic layer was further washed three times with NaHCO_3_ solution. Subsequently, the organic layer was washed with 20 mL of 26% saturated sodium chloride (NaCl) and 20 mL of distilled water twice to avoid formation of emulsion. Once the two layers were formed, the bottom aqueous layer was removed. The washing process was repeated until the organic layer with pH 7 was obtained. Then, the sample was poured into a round flask and connected to a rotary evaporator apparatus (90–100 °C) to remove any excess toluene and unreacted alcohol. The remaining water in the sample was absorbed by sodium sulfate (Na_2_SO_4_) over night and filtered off. The organic product was rotary-evaporated to remove ethyl acetate at 80 °C, giving a viscous semi-solid polyolester.

### 2.4. Lubrication properties analysis

The American Society for Testing Materials standards (ASTM) was used to determine the lubrication characteristics. For certain, ASTM D-6186 of oxidative stability [17], ASTM D-97 of pour point [18] and ASTM D-93 of flash point [19]. The oxidative stability temperature (OS_T_) in the current study was carried out using pressurized differential scanning calorimetry (PDSC822e, Metter Toledo), which is a highly-selected device to evaluate the OS_T_ of oleochemicals. Rheometer with model physical MCR 301 from Anton Paar Instruments (Germany) was used to measure kinematic viscosity. The kinematic viscosity and viscosity index were calculated according to ASTM method ASTM D 2270-93 [20]. All the measurements were performed in triplicate and the data was reported as a mean ± SD of triplicate determinations.

### 2.5. Statistical analysis and experimental design

To assess the reaction of the synthesized biolubricat, this study employed a three-factor D-optimal design. The independent variables were denoted as X_1_ for H_2_SO_4_ (%) concentration (as a percentage of the weight of fatty acid), X_2_ for esterification temperature (°C) and X_3_ for esterification time (h). The low value (−1) and high value (+1) of X_1_, X_2_, and X_3_ as can be seen from Table 2 were equivalent with the range setting of each parameter: 1%–5% for X_1_, 2–6 h for X_2_ and 110–150 °C for X_3_. Biolubricant yield Y_1_ (%) and concentration of triester Y_2_ (%) were determined using the D-optimal experimental design generating 18 experiment runs, as presented in Table 3.

## 3. Results and discussion

### 3.1. Esterification of SFA-PFAD with polyhydric alcohol

**Figure 1:**
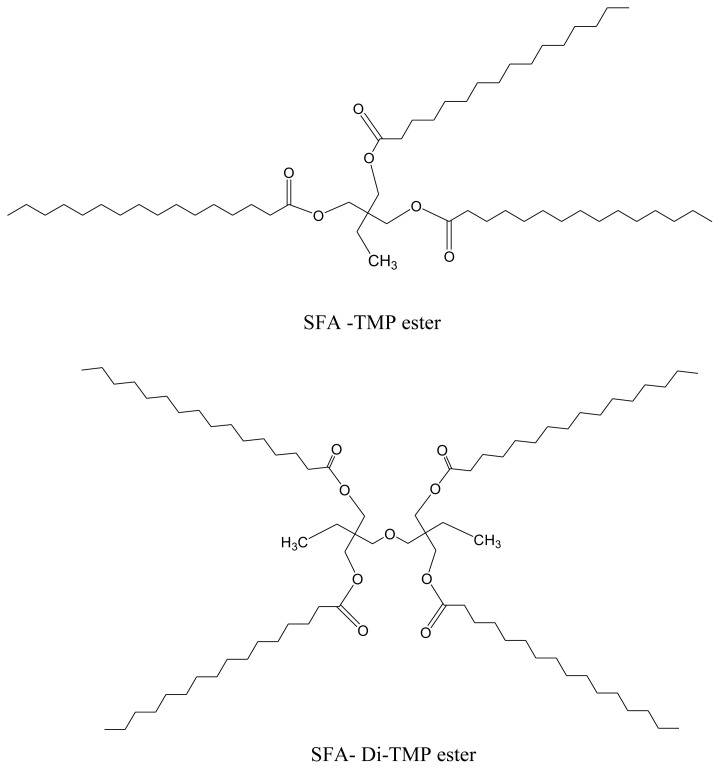

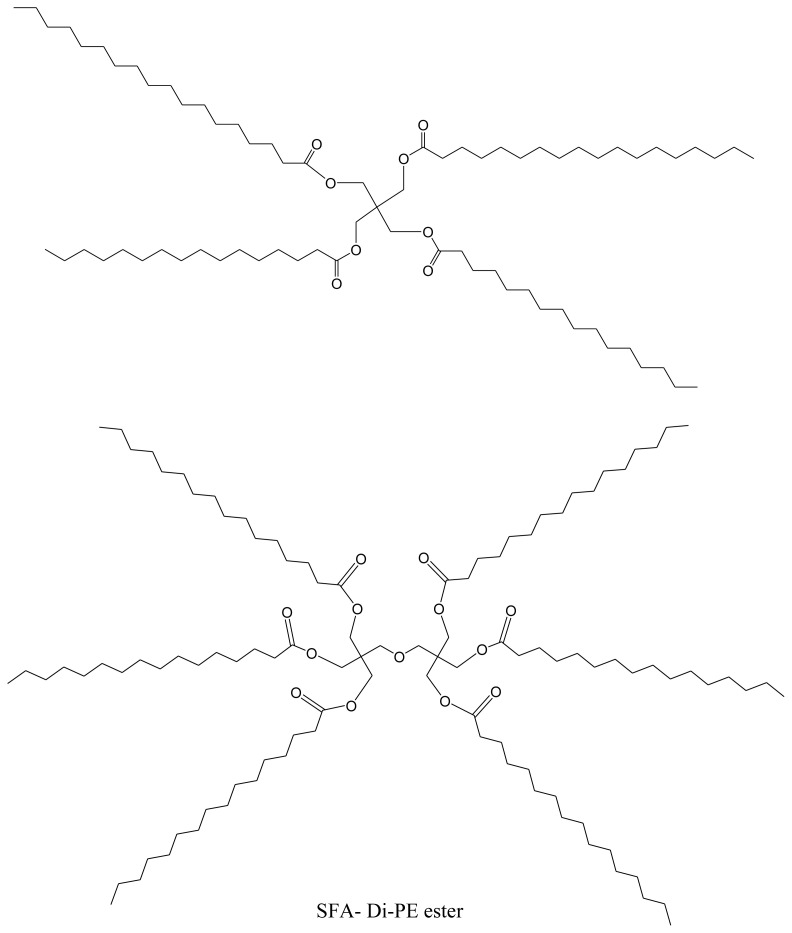
Chemical structure of the synthesized esters.

Saturated palm fatty acids distillate (SFA-PFAD) was mixed separately with four high degree polyhydric alcohols during the esterification process to produce four SFA-PFAD-based esters known as saturated palm fatty acid distillate-trimethylolpropane (SFA-TMP) ester, saturated palm fatty acid distillate-di-trimethylolpropane (SFA-Di-TMP) ester, saturated palm fatty acid distillate-pentaerythritol (SFA-PE) ester and saturated palm fatty acid distillate-dipentaerythritol (SFA-Di-PE) ester as shown in Figure 1.

### 3.2. Response surface methodology optimization

The optimum condition and high percentage of yield production were determined using response surface methodology (RSM). The interaction between response variables and other variable influences can be assessed using RSM. Response surface methodology aims to decrease the number of experimental runs required to obtain satisfactory information for statistically agreeable results [21]. Consequently, it reduces time-consuming and protracted analyses to full-factorial experimentation. Table 2 shows the use of D-optimal design for varying the independent variables and their levels as a different acid catalyst, with different temperatures and times for esterification between SFA-PFAD and TMP for an example of the optimization. The D-optimal design is a statistical program offered by a computer algorithm. When classical designs are ineffective, they can be replaced by these types of computer-aided designs.

The three-factor D-optimal design was used to study the percentage of OH, the yield of SFA-TMP ester, and the percentage of tri-ester in SFA-TMP ester. The effects of different process conditions on the responses were optimized and determined using RSM. The response factors and values were determined via an initial screening step. In this step, X_1_, X_2_, and X_3_ were used to represent the acid catalyst (mL/g), time (h), and temperature (°C), respectively. Each variable was evaluated by varying their values within a minimum (−1) and maximum (+1) value, as presented in Table 2. The experimental data of the yield of SFA-TMP ester and tri-ester percentage of all the samples are provided in Table 3.

### 3.3. Model fitting of D-optimal design

The D-optimal design from the Design-Expert v. 6.0.10 (Stat-Ease, USA) software was applied for the esterification optimization process. Three independent variables, acid catalyst (X_1_), esterification time (X_2_), and esterification temperature (X_3_), were used to conduct the RSM. This is because these variables affected the yield of SFA-TMP ester % (Y_1_), and tri-ester % (Y_2_). D-optimal design analysis showed a total of 18 experiments, which is required to estimate the


(1) $${\text{Y}}_{1}=+82.83+3.36\;{\text{X}}_{1}+3.21\;{\text{X}}_{2}+2.20\;{\text{X}}_{3}-0.3022\;{\text{X}}_{1}{\text{X}}_{2}-1.06\;{\text{X}}_{1}{\text{X}}_{3}+0.3927\;{\text{X}}_{2}{\text{X}}_{3}+3.87\;{{\text{X}}_{1}}^{2}+0.5355\;{{\text{X}}_{2}}^{2}-1.99\;{{\text{X}}_{3}}^{2}.$$

(2) $${\text{Y}}_{2}=+54.39+13.65\;{\text{X}}_{1}+14.79\;{\text{X}}_{2}+9.50\;{\text{X}}_{3}-2.00\;{\text{X}}_{1}{\text{X}}_{2}-6.00\;{\text{X}}_{1}{\text{X}}_{3}+3.39\;{\text{X}}_{2}{\text{X}}_{3}-2.74\;{{\text{X}}_{1}}^{2}+4.95\;{{\text{X}}_{2}}^{2}+12.08\;{{\text{X}}_{3}}^{2}.$$

coefficients of all models by using a quadratic polynomial regression model. A multifaceted relationship between the independent variables (X_1_, X_2_, and X_3_) includes both first- and second-order polynomials, as observed in Equations 1 and 2 for the yield % (Y_1_), tri-ester % (Y_2_) in the SFA-TMP ester.

The regression coefficients and analysis of variance (ANOVA) of the model for the yield and tri-ester percentage are listed in Tables 4 and 5, respectively. The independent variables were used to obtain the R-squared values, which measures the amount of reduction in the variability of responses, where the high R^2^ correlation value of the model indicates good fitting. Also, the adjusted R-squared correlation can be utilized to determine the fit of a regression model [22]. The model was highly significant regarding Y_1_, and Y_2_, with R-squared values of 0.9751, and 0.9520, respectively. In this study, R-squared values for all responses show a good correlation between the predicted values and the actual results of the dependent variables derived from the model.

Furthermore, as regards the yield % of ester (Y_1_), the R-square value of 0.9751 shows that the model is capable of explaining about 97.5% of the variation on the response, and the model did not describe only 2.5% of the variations. The adjusted R-squared value (adj. R-squared = 0.9471) shows that the model is significant, and its value is slightly close to the R-squared value of 0.9751. Also, regarding the percentage of tri-ester (Y_2_), the R-square value of 0.9520 shows that the model is capable of explaining about 95% of the response variation. Conversely, the total variations that the model could not describe were only 5%. The model is significant, as proven from the adjusted R-squared value (adj. R-squared = 0.8981) that is slightly close to the R-squared value of 0.9520.

The linear effect of an acid catalyst (X_1_) was highly significant (p < 0.01) for a percentage of yield (Y_1_), and the percentage of tri-ester (Y_2_). The linear effect of esterification time (X_2_) was also highly significant (p < 0.01) for a percentage of yield (Y_1_) and tri-ester (Y_2_). The linear effect of esterification temperature (X_3_) was highly significant (p < 0.01) for the yield of ester (Y_1_) and tri-ester (Y_2_).

The percentage of tri-ester % (Y_2_) varied significantly from 29% to 99% when the acid catalyst (X_1_), time (X_2_), and temperature (X_3_) were varied, as given in Table 5. From the model’s ANOVA results, the effect of an acid catalyst (X_1_), time (X_2_), and temperature are shown to strongly affect the percentage of tri-ester (Y_2_), where a highly significant (p < 0.01) was observed for the X_1_, X_2_, and X_3_ quadratic term on Y_2_. Besides, there was no significant (p < 0.05) interaction effect between acid catalyst (X_1_) and time (X_2_) on the percentage of tri-ester (Y_2_) and the interaction effect between time (X_2_) and temperature (X_3_) on the percentage of tri-ester (Y_2_), indicating that Y_2_ is not significantly affected by these factors.

### 3.4. Adequacy check of the model

**Figure 2:**
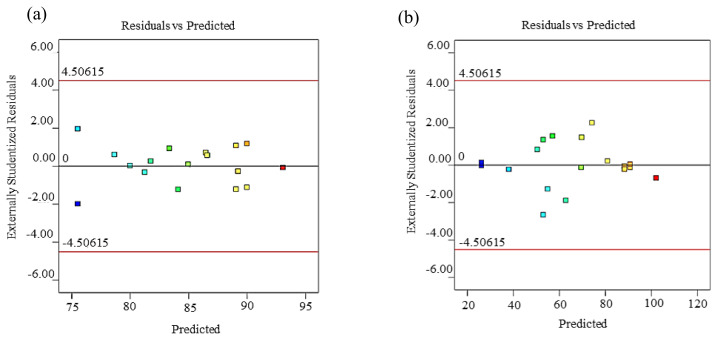
Studentized residuals against predicted value for the percentage of the yield of ester (a) and tri-ester (b).

The data analysis of the model must be confirmed by an adequacy check examination to guarantee that the model accurately predicts the real system or determine if the analysis may give unreliable or poor results [23]. The studentized residuals are plotted versus the predicted response for the yield (Y_1_) and tri-ester (Y_2_) as shown in Figures 2a and 2b, respectively. The apparent difference observed in the first examinations in the graph is an arbitrary diffusion, which demonstrates that the difference is steady for all response values. In the event that the difference of the response depends mainly on the mean level of Y, then a dissipate plot will regularly be demonstrated a funnel-shaped pattern [24], which is the case for this study and there were no outlier’s data and clustering by the group in the data also.

**Figure 3:**
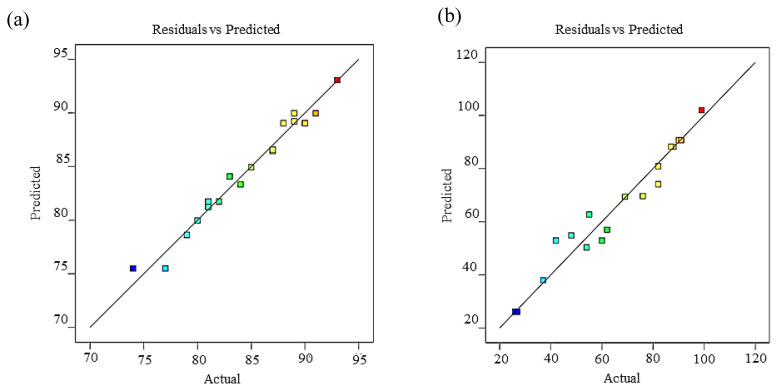
Plot between actual (experimental) against predicted of the percentage of the yield (a), and tri-ester (b).

On the other hand, the predicted responses and the actual (experimental) responses were in reasonable conformity. The predicted response values were obtained from the model, while the values of the actual response are calculated from the experimental runs. The relationship is illustrated by plotting the graph of predicted responses against actual responses. A linear line was obtained, indicating that this model provided a good approximation of the experimental response as given in Figures 3a and 3b for a percentage of the yield of ester and tri-ester, respectively.

### 3.5. Response surface analysis and optimization conditions

The model equations (1) and (2) were used to construct the three-dimensional (3D) response surface for the percentage of the yield (Y_1_) and tri-ester (Y_2_). In these ways, the interaction between variables could be visualized. Besides that, the optimal value of the maximum percentage of yield (Y_1_) and the maximum percentage of tri-ester (Y_2_) via esterification reaction of SFA-PFAD with TMP can be verified. In the 3D graphs, the interaction between the two variables was plotted, whereas another factor was kept constant at its central level. The central values were 3% of an acid catalyst, 4 h of esterification time, and 130 °C of esterification time.

**Figure 4:**
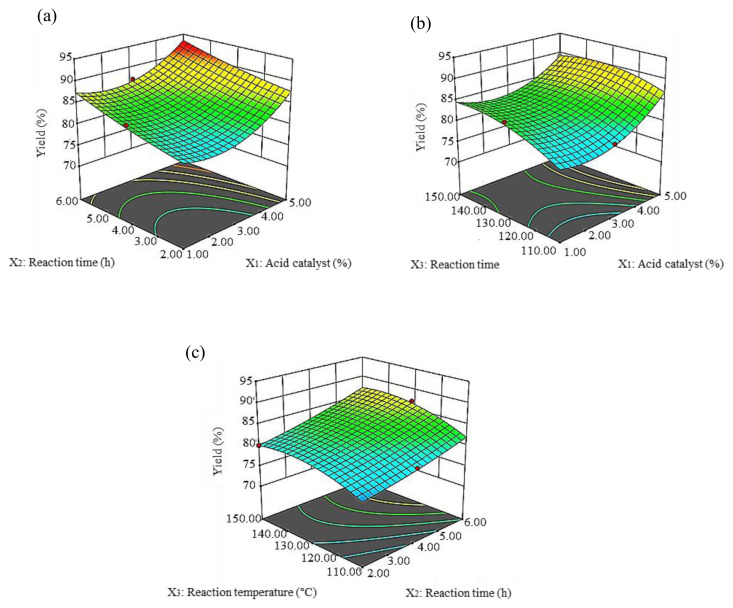
Three-dimensional response surface (3D) of the yield percentage (Y_1_) as a function of an acid catalyst (X_1_, %) and time (X_2_, h) (a), acid catalyst (X_1_, %) and temperature (X_3_, °C) (b), time (X_2_, h), and temperature (X_3_, °C) (c).

**Figure 5:**
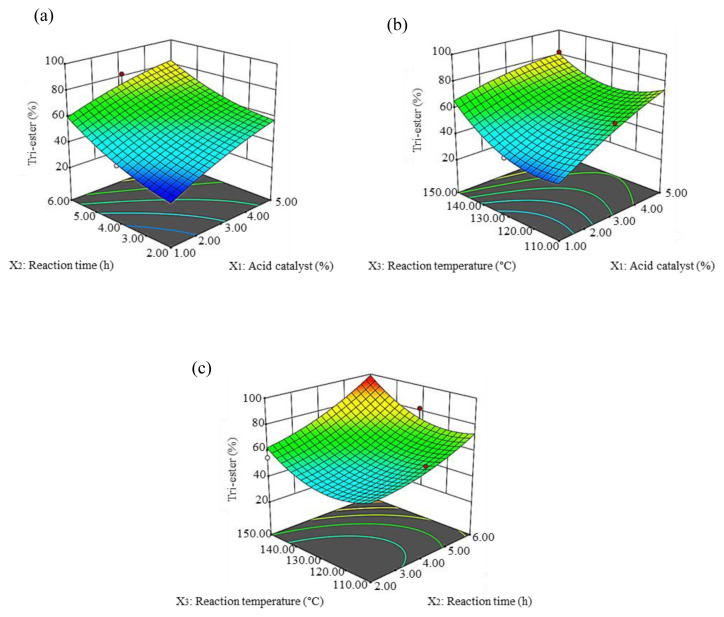
Three-dimensional response surface (3D) of tri-ester percentage (Y_2_) as a function of acid catalyst (X_1_, %) and time (X_2_, h) (a), acid catalyst (X_1_, %) and temperature (X_3_, °C) (b), time (X_2_, h), and temperature (X_3_, °C) (c).

The interaction effect between acid catalyst and time is presented in Figure 4a. The maximum yield percentage at 93% was obtained at an acid catalyst of 5% and time 6 h. The 3D graph shows that the maximum yield percentage was obtained at an acid catalyst of 5% and a temperature of 150 °C (Figure 4b). The combined effect of time and temperature demonstrated that ester yield increased with an increase in time (Figure 4c). The interaction effect between the acid catalyst and time on the tri-ester percentage of SFA-TMP ester is shown in Figure 5a. The 3D plot shows that increasing the ratio of acid catalyst from 1%–5% continuously improved the tri-ester percentage of SFA-TMP ester, while the optimal time for the maximum tri-ester was at 6 h. The effect of acid catalyst and temperature led to an increase in the tri-ester with an increase in acid catalyst and temperature (Figure 5b). The effect of time and temperature also resulted in an increase of tri-ester with an increase in the acid catalyst and temperature (Figure 5c).

### 3.6. Model validation and experimental confirmation

**Figure 6:**
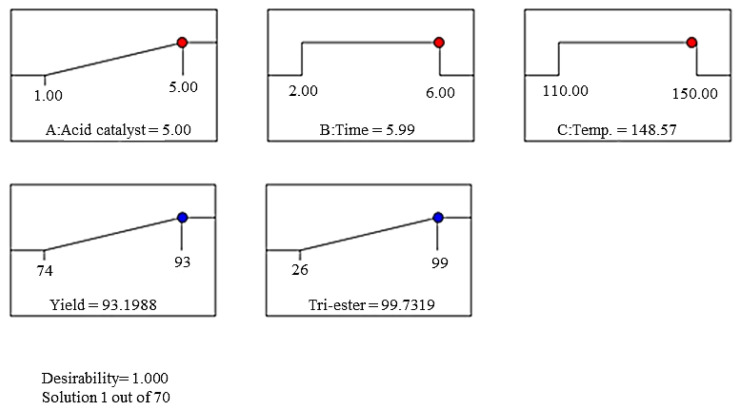
Predicted conditions to produce desirable results of dependent variables using D-optimal design.

The desirability function was used to investigate the validity of predicted models developed to determine the optimal conditions for the response variables: the yield of ester and tri-ester percentage with maximum values as shown in Tables 6, 7, and Figure 6. The validity of the predicted model was tested via a validation test. A triplicate investigation was conducted under optimum conditions, comprising acid catalysts of 5%, esterification time 6 h, and esterification temperature of 150 °C. The yield was approximately 93.1 ± 0.5% with 99 ± 0.9 % of tri-ester selectivity were obtained at the optimal esterification condition for SFA-TMP ester. The esterification reaction efficiency has been monitored by measuring the hydroxy, OH value for the ester products at selected particular reaction time as shown in Table 8.

The ester final products have shown the progress and the efficiency of the esterification process have reached up to the range of 80%–88% completion. A simple and less bulky molecular structure of the ester (SFA-TMP) has shown highest completion percentage (88%) of esterification process compared to other ester products. This is due to SFA-TMP has the less steric hindrance effect to ease the combination/reaction between the substrates compare to the more complex structure such as of SFA-Di-PE ester (80%). Never the less, the overall ester products yield has been significantly increased after the optimization process by using D-optimal design as compare to the conventional factor by factor optimization method as shown in Table 9.

**Figure 7:**
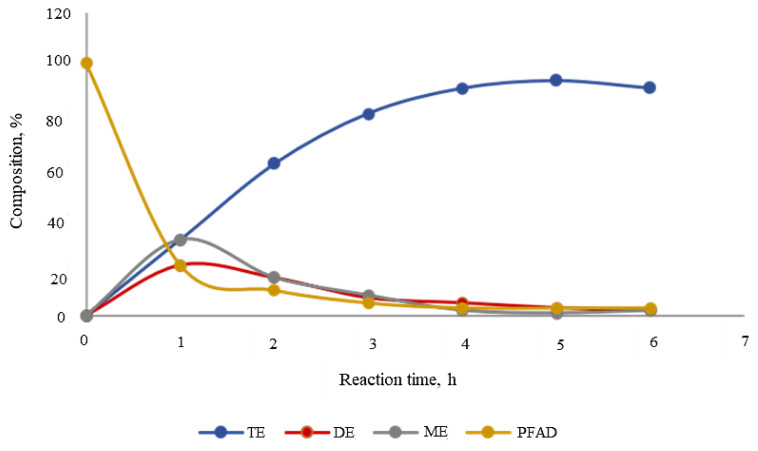
Esterification between SFA-PFAD and TMP at optimal condition of 150 °C and 3.5:1 molar ratio at 6 h using 5 wt% H_2_SO_4_ catalyst.

Time line esterification also has been monitored toward the completion of the final tri-ester SFA-TMP, as an example for the discussion. Figure 7 indicates the progress of esterification reactions at different times at the esterification optimal condition. It was observed that esterification proceeded stepwise, in which SFA-TMP mono-ester (ME) formation first reached a maximum value. This was followed by a steady formation of SFA-TMP di-ester (DE). At the point of maximum formation of DE, SFA-TMP tri-ester (TE) increased rapidly. This was because the esterification stepwise process preceded with the formation of intermediates products, before the commencement of the formation of final desired SFA-TMP tri-ester product. Initially, ME, which was a single branch polyol ester was formed during the reaction. The increasing amount of ME, however, would immediately undergo conversion to form DE, which would react with SFA-PFAD to produce SFA-TMP tri-ester. Concentration of SFA-TMP tri-ester (TE) would rise with the decrease of DE and ME concentrations.

**Figure 8:**
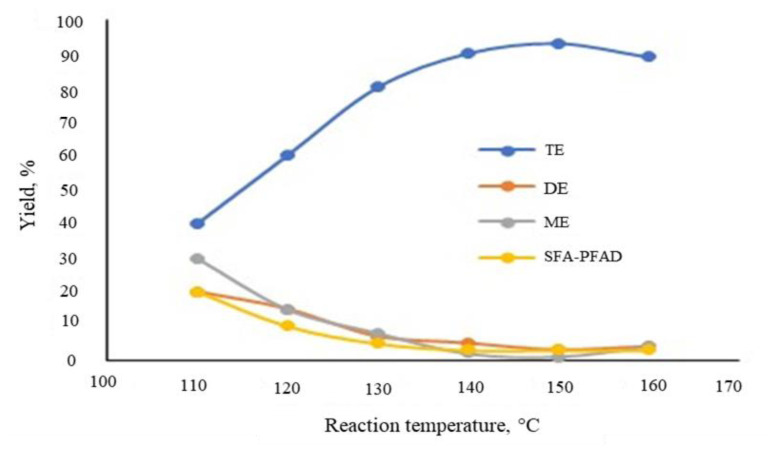
Effects of temperature on SFA-TMP ester composition at 3.5:1 SFA-PFAD:TMP mole ratio and 5% catalyst loading for 6 h.

To determine the effect of temperature, a series of experiments was conducted at SFA-PFAD:TMP molar ratios of 35:1, catalyst (H_2_SO_4_) amount was fixed at 5.0% wt/wt of reaction mixture. The reactions monitored for fixed 6 h were carried out at 110, 120, 130, 140, 150, and 160 °C to observe the effect of temperature on the esterification products. Figure 8 shows the influence of temperature on the production profile of SFA-TMP consisting of mono-ester (ME), di-ester (DE), tri-ester (TE), and unreacted SFA-PFAD. Figure 8 indicated that as the temperature increased, the TE composition increased, until at about 150 °C, after which the increase in TE composition became marginal. This was because at higher temperature (>150 °C), the amount of SFA-PFAD was low as a result of vaporization and decrease the TE conversion. The amount of TE would be considered insignificantly unchanged with temperature variation above 150 °C.

### 3.7. Esters structural characterization

**Figure 9:**
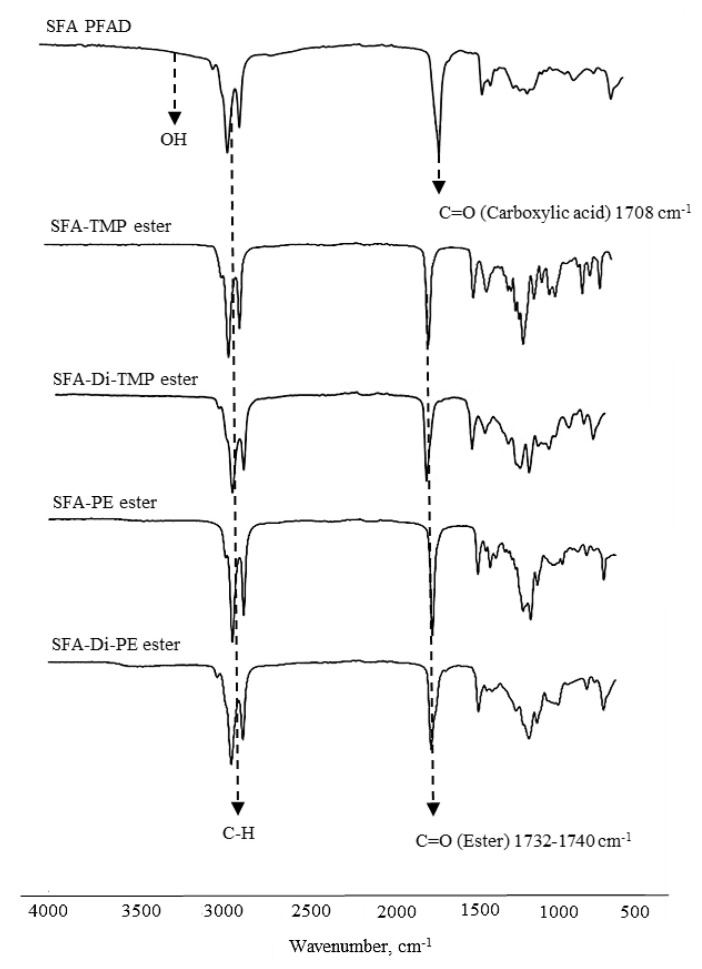
FTIR spectra of SFA-PFAD and synthesized SFA-PFAD-based esters.

The final products of the synthesized SFA-PFAD esters were characterized and analyzed using FTIR, ^1^H, ^13^C NMR, and high temperature column GC-FID. The chemical structure of optimized synthesized SFA-PFAD esters after RSM was verified using FTIR and NMR (^1^H and ^13^C) spectroscopy. FTIR analyses were carried out to verify the esters and confirm the success of the esterification reaction. The FTIR spectra of SFA-PFAD esters (SFA-TMP, SFA-Di-TMP, SFA-PE, and SFA-Di-PE) were similar. Strong intensities were observed corresponding to C=O and C-O of ester, stretching vibration of the carbonyl group (C=O) for aliphatic esters present in the wave number range from 1732–1740 cm^−1^, while in the SFA the C=O stretching vibration of carboxylic acids present in wave numbers 1690–1725 cm^−1^. Table 10 displays the main absorption peaks of the functional groups for all the synthesized SFA-PFAD esters. From the FTIR spectra, we can see that the stretching vibration of the carbonyl group (C=O) for aliphatic esters is in wave number 1732, 1738, 1737 and 1740 cm^−1^ for SFA-TMP ester, SFA-Di-TMP ester, SFA-PE ester and SFA-Di-PE ester respectively. The peaks at 1172 cm^−1^ and 1240 cm^−1^ denote the C-O stretching bands of the ester functional group. It can be observed that the unreacted alcohol and SFA-PFAD had disappeared from the final product (ester) from the spectra showing that there were no stretching vibrations of alcohol (OH) and no bond stretching of acids (-COOH) as shown in Figure 9.

**Figure 10:**
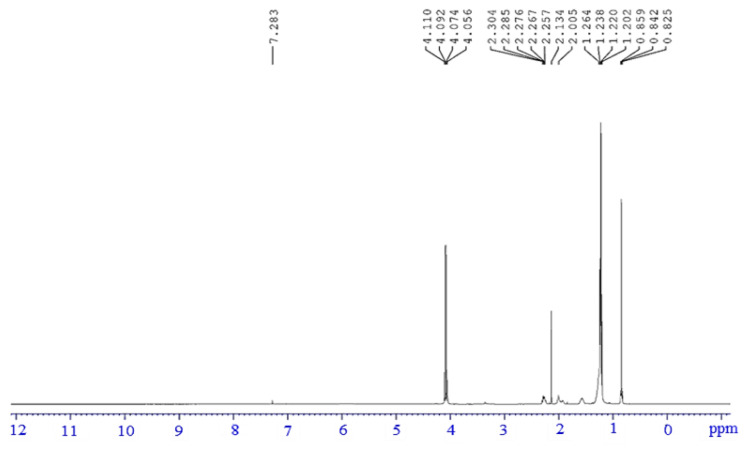
^1^H NMR spectrum of SFA-TMP ester.

The ^1^H spectrum of SFA-TMP ester is indicated by Figure 10. The disappearance of the ^1^H chemical shift of the proton (H) of the carboxylic acid group (-COOH) at 11.0 ppm, and for the alcohol group (-OH) at 4.7 ppm, confirmed the success of the esterification reaction [25]. ^1^H chemical shift ranges for aliphatic protons (-CH_2_) were detected, normally at about 1.25–1.56 ppm. However, the two protons of the -CH_2_-O-C=O ester group shift appeared at about 3.80–4.01 ppm [26]. The saturated fatty acids mixture of palmitic acid, stearic acid, and myristic acid compose the SFA-TMP ester. Alkene groups were identified to determine whether or not unsaturated fatty acids were present. The methylene proton signal (-CH-CH-) was disappeared at 5.32–5.38 ppm, signifying the unsaturated fatty acids was absence in SFA-TMP esters [27].

**Figure 11:**
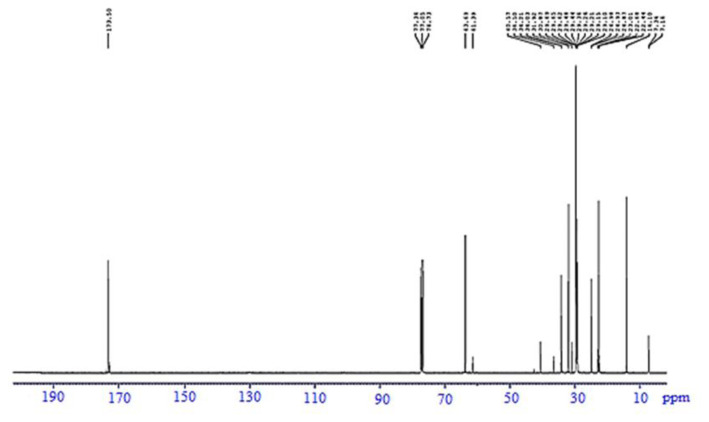
^13^C NMR spectrum of SFA-TMP ester.

The ^13^C NMR spectrum of SFA-TMP ester is shown in Figure 11. The signal at 171.09–174.07 ppm indicates the carbon ester carbonyl (C=O) in SFA-TMP ester. Furthermore, a peak at 60.32–64.14 ppm attributed to -CH_2_-O-CO-R group. Also, a signal at 40.57–42.44 ppm was found to correspond to the carbon atom in TMP (-CH_2_-COOR). The chemical shift of the quaternary carbon occurs within 20–60 ppm and this is in agreement with the results of the study Pavia et al. [27]. The carbon atoms of -CH_2_ were identified from the peaks at 22.16–36.46 ppm, denoting saturated alkyl chain a common indication of the presence of these compounds [28,29]. The terminal methyl (-CH_3_) peak appeared at 14.04–14.08 ppm.

### 3.8. Lubrication properties of SFA PFAD esters

The lubrication properties of the synthesized SFA PFAD esters (SFA-TMP, SFA-Di-TMP, SFA-PE, and SFA-Di-PE) were tested. These properties include OS_T_, flash point (FP), pour point (PP), and viscosity index (VI) as shown in Table 11.

#### 3.8.1. Viscosity index

The more viscose the oil, the more it will be able to reduce wear and friction. Viscosity is highly prized parameter in lubricants for the automotive industry. The viscosity properties of the oil either in cold or hot temperatures should not hinder or must be able to let the engine components ‘glide’ over each other [30]. To determine how viscosity changes with temperature, one should refer to its viscosity index, VI [31]. A low VI represents relatively large changes in kinematic viscosity induced by changes in temperature and vice versa and should be avoided [32].

Table 11 presents the values of the viscosity index of the synthesized SFA-PFAD esters. The results prove that the kinematic viscosity and VI values increased with the carbon-chain length of the SFA and alcohols used. The viscosity index results in Table 10 highlight the importance of selecting raw materials for the esterification reaction to produce esters with high values of the viscosity index. From Table 10, SFA-PFAD esters with high molecular weight have the most promising criteria to be used as a biolubricant with high kinematic viscosity and VI value compared to other synthesized esters dodecanedioate ester (DHD) [33,34].

#### 3.8.2. Pour point

The oil that is to be used as biolubricant must have a key property at low temperatures, which is a low pour point, PP. Lubricant PP is defined as the least temperature in which oil will not turn solid (i.e., the liquid still behaves like a fluid). The PP of the synthesized SFA-PFAD esters is summarized in Table 11. All SFA-PFAD esters are semi-solid to solid at room temperature. From the PP results, it can be concluded that SFA PFAD esters, which were constructed from SFA-PFAD and alcohols, have resulted in high PP values compared to unsaturated carbons chain fatty acid. The order of the ester PP is arranged as follows: SFA-TMP ester > SFA-PE ester > SFA-Di-TMP ester > SFA-Di-PE ester. The respective PP values of these esters are 35 ± 2 °C, 33 ± 2 °C, 30 ± 2 °C, and 25 ± 2 °C, respectively.

These results appeared that the PP of esters depends on the chemical structure and weight of alcohols used. Therefore, it is important to carefully select the raw material to be esterified to produce high-quality lubricant. A high PP correlates to a synthesized SFA-PFAD ester with high molecular weight, owing to its saturated aliphatic carbon atoms (C-C), which could be the factor for the high PP of SFA-PFAD esters.

#### 3.8.3. Flash point

The flash point, FP of the oil is the lowest temperature at which it can vaporize to form an ignitable mixture in the air. The FP of oil is an important safety property as it refers to the lowest temperature at which the auto-ignition of the vapor occurs above the heated oil sample. Salimon and Salih [33] indicated this as a key factor that will determine the potential biolubricant’s behavior. The system condition, design, and operational method are what determine the FP value. It is easy to select the temperature for storing the oil, and which transportation type to use if the FP of the oil is known. Biolubricant producers draw possible combinations of products by looking at their FP. For instance, extra precautions and safe handling would be required with low FP biolubricants, as these are probably contaminated with volatile products [35,36]. Table 10 shows the FP values of the synthesized SFA-PFAD esters.

The results in Table 11 indicate that the FP values increase among the esters with higher molecular weight. SFA-Di-PE ester with high in molecular weight have the highest flash points in the range of 310 ± 5 °C, followed by SFA-Di-TMP ester with 290 ± 5 °C, SFA-PE ester with 275 ± 5 °C and SFA-TMP ester with 270 ± 5 °C, respectively. These esters are acceptable for commercial uses, which are comparative to commercial biolubricant with a high FP value over of 200 °C [31].

Generally, the tendency of a substance to vaporize is dependent on the strength of intermolecular forces. For SFA-PFAD esters having intermolecular forces along the saturated straight carbon chain length are strong, and molecules tend to stick firmly. Therefore, SFA-PFAD ester requires higher energy before breaking bonds and for volatile molecules to form a combustible mixture [3]. The strong dipole moments (London forces) binding the ester group in SFA esters together promote reduced volatility and raises the FP, which makes it better than mineral oils [29].

#### 3.8.4. Oxidative stability

Oxidative stability is an important lubrication property that measures the resistance of biolubricant and fuel to oxidation. As the process takes place through a chain reaction, oxidation has a period where the reaction is relatively slow before it suddenly proliferates. Pressure differential scanning calorimetry (PDSC) is an effective tool for measuring oxidative stability by determining the OS_T_ (°C) of a biolubricant in an accelerated mode. The OS_T_ is defined as the temperature at which the oxidation rate increased sharply, observed, and obtained from the extrapolation of the tangent line on the steepest slope of the plot of the exothermic reaction heat flow versus temperature. The more stable the biolubricant to oxidative, the higher the OS_T_ [37].

The synthesized SFA-PFAD esters were scanned to measure their OS_T_ using PDSC. The results from each scan were analyzed to determine the OS_T_. Loss of mass in the sample shows up as split peaks, baseline changes, and tailing, and manifests as evaporation. This was more evident, however, when temperatures went beyond the starting oxidation peak. Heat flow (W/g) of each ester was plotted against temperature to determine ester OS_T_.

Table 11 presents the OS_T_ of tested SFA-PFAD esters. The results show the OS_T_ of SFA-PFAD esters constructed from polyhydric alcohols are as follows: SFA-TMP ester (322 ± 2 °C); SFA-Di-TMP ester (260 ± 2 °C); SFA-PE ester (300 ± 2 °C), and SFA-Di-PE ester (251 ± 2 °C), respectively. These products are more stable than the USFA PFAD esters and higher oxidative stability, which contributes to increasing the intermolecular forces of hydrogen bonding, London forces among the saturated carbon chain -CH_2_-[38].

### 3.9. GC-FID analysis

**Figure 12:**
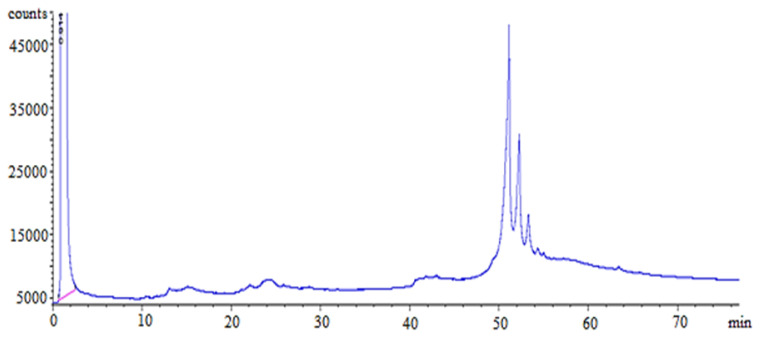
GC-FID chromatogram of SFA-TMP ester.

The final step to confirm the chemical structure of the synthezied ester was analyzed using the high column temperature GC. When the SFA-PFAD and TMP are reacted to synthesized SFA-TMP ester, the tri-ester product should be determined. The terminology used in fats and oils analysis and the TMP skeleton-attached alkyl group carbon number was referred to identify the tri-ester peaks. The results show a 99.9% major tri-ester was produced. Figure 12 shows tri-ester peaks statting to form at retention time (RT) of 51.20 min, indicating the successful conversion of the TMP OH group to TMP ester group. The tri-esters selectivity of SFA-TMP ester was increased from 90%–99.9% after the applied D-optimal design of response surface methodology (RSM). The observation shown that the RSM statistical analysis has a significant effect on the operating condition of the esterification reaction to increasing tri-ester composition. The same effect of D-optimal design into the yield percentage of ester was also increased from 86%–93%, as shown before in Tables 6 and 7. There for the D-optimal RSM optimizations have high efficiency in selecting the optimum condition for the esterification reaction.

## 4. Conclusion

The saturated palm fatty acid distillate-polyhydric alcohol-based esters have successfully synthesized. The esterification of SFA-PFAD with TMP was optimized based on the D-optimal approach for RSM optimization method. The effects of the interactions between independent variables on the SFA-TMP esterification were investigated with the ANOVA results confirming a consistency between the predictions from the model and the experimental data. The optimum conditions for the esterification of SFA-TMP at mole ratio of 3.5:1 was obtained at acid catalysts of 5%, esterification time and temperature of 6 h and 150 °C respectively. The highest yield percentage of SFA-TMP (93 ± 0.5%) with high selectivity of tri-ester (99 ± 0.9%) was observed at the optimal condition. The findings prove that the prediction model for the esterification of SFA-TMP ester from saturated palm fatty acid distillate has been validated. Despite their high PP, other lubrication properties such as VI, OS_T_, and FP are comparable to dodecanedioate ester industrial biolubricant. In general, the results are indicative that the SFA PFAD esters produced have good lubrication properties. SFA-PFAD esters promising for special and environmentally sensitive applications and can be used as biolubricants in applications such as machinery, chainsaw, and dust-suppressant fluids in a tropical country.

## Figures and Tables

**Table 1:** The amount of saturated palm fatty acid distillate (SFA-PFAD) and polyhydric alcohol for the esterification reactions.

Ester	Mole	
	SFA	Alcohol
SFA-TMP	3.5	1
SFA-Di-TMP	5	1
SFA-PE	5	1
SFA-Di-PE	7	1

Notes: SFA-TMP = Saturated palm fatty acid distillate-trimethylolpropane ester; SFA-Di-TMP = Saturated palm fatty acid distillate-di-trimethylolpropane ester; SFA-PE = Saturated palm fatty acid distillate-pentaerythritol ester; SFA-Di-PE = Saturated palm fatty acid distillate-di-pentaerythritol ester.

**Table 2:** Independent variables and levels of D-optimal design for the esterification reactions.

Independent variables	Factor X_i_	Variable levels		
		−1	0	1
H_2_SO_4_ concentration (%, w/w)	X_1_	1	3	5
Esterification time (h)	X_2_	2	4	6
Esterification temperature (°C)	X_3_	110	130	150

**Table 3:** Experimental runs of D-optimal design and response for the esterification reactions.

Run	Variables levels, X			Responses, Y	
	X_1_	X_2_	X_3_	Y_1_	Y_2_
1	5.00	4.00	150.00	89	82
2	5.00	2.00	150.00	87	76
3	1.00	6.00	110.00	81	60
4	5.00	2.00	110.00	85	69
5	1.00	6.00	150.00	90	90
6	3.00	4.00	110.00	79	62
7	5.00	6.00	110.00	89	87
8	1.00	2.00	150.00	81	54
9	3.00	6.00	130.00	87	82
10	1.00	2.00	110.00	77	27
11	4.00	3.00	130.00	83	48
12	5.00	6.00	150.00	93	99
13	1.00	4.00	130.00	84	37
14	3.00	2.00	150.00	80	55
15	1.00	6.00	110.00	82	42
16	5.00	6.00	110.00	91	88
17	1.00	6.00	150.00	88	91
18	1.00	2.00	110.00	74	26

Notes: X_1_= Acid catalyst (%); X_2_ = Esterification time (h); X_3_ = Esterification temprature (°C); Y_1_ = Yield (%); Y_2_ = Tri-ester (%).

**Table 4:** Analysis of variance (ANOVA) of yield percentage of ester (Y_1_).

Source	Sum of squares	Degree of freedom	Mean square	F-value	P-value	Status
Model	448.99	9	49.89	34.84	< 0.0001	Significant
X_1_	144.85	1	144.85	101.14	< 0.0001	
X_2_	132.10	1	132.10	92.24	< 0.0001	
X_3_	68.92	1	68.92	48.12	0.0001	
X_1_X_2_	1.02	1	1.02	0.7124	0.4232	
X_1_X_3_	13.54	1	13.54	9.46	0.0152	
X_2_X_3_	1.87	1	1.87	1.31	0.2856	
X_1_^2^	31.47	1	31.47	21.98	0.0016	
X_2_^2^	0.6037	1	0.6037	0.4215	0.5344	
X_3_^2^	6.78	1	6.78	4.74	0.0612	
Residual	11.46	8	1.43			
Lack of fit	2.46	4	0.6141	0.2729	0.8818	Not significant
Pure error	9.00	4	2.25			
Cor total	460.44	17				

Notes: R^2^ = 0.9751; R^2^adj = 0.9471; Adequate precision = 19.6752; X_1_ = Acid catalyst (%), X_2_ = Esterification time (hr); X_3_= Esterification temperature (°C).

**Table 5:** Analysis of variance (ANOVA) of tri-ester percentage (Y_2_).

Source	Sum of squares	Degree of freedom	Mean square	F-value	P-value	Status
Model	8573.71	9	952.63	17.65	0.0002	Significant
X_1_	2388.39	1	2388.39	44.24	0.0002	
X_2_	2802.92	1	2802.92	51.92	< 0.0001	
X_3_	1280.91	1	1280.91	23.73	0.0012	
X_1_X_2_	44.74	1	44.74	0.8288	0.3892	
X_1_X_3_	437.69	1	437.69	8.11	0.0216	
X_2_X_3_	139.37	1	139.37	2.58	0.1468	
X_1_^2^	15.83	1	15.83	0.2933	0.6029	
X_2_^2^	51.58	1	51.58	0.9554	0.3570	
X_3_^2^	248.90	1	248.90	4.61	0.0641	
Residual	431.90	8	53.99			
Lack of fit	268.40	4	67.10	1.64	0.3214	Not significant
Pure Error	163.50	4	40.88			
Cor total	9005.61	17				

Notes: R^2^ = 0.9520; R^2^adj = 0.8981; Adequate precision: 13.8535; X_1_ = Acid catalyst (%); X_2_ = Esterification time (h); X_3_ = Esterification temperature (°C).

**Table 6:** Optimization criteria for dependent variables.

Variables	Goal	Lower limits	Upper limits
Acid catalyst (%) (X_1_)	in the range	1	5
Esterification time (h) (X_2_)	in the range	2	6
Esterification temperature (ºC) (X_3_)	in the range	110	150
Yield (%), (Y_1_)	Maximize	74	93
Tri-ester (%), (Y_2_)	Maximize	29	99

**Table 7:** Result of model validation at the optimum conditions (verification test).

Factors	Variables			Responses	
	X_1_	X_2_	X_3_	Y_1_	Y_2_
Predicted	5	6	150	93.05	98.52
Actual	5	6	150	93 ± 0.5	99 ± 0.9
Manual optimization	2	6	150	86 ± 0.5	90 ± 0.5

Notes: X_1_ = Acid catalyst (%); X_2_ = Esterification time (h); X_3_ = Esterification temperature (ºC); Y_1_ = Yield (%); Y_2_ = Tri-ester (%).

**Table 8:** The products OH value after the esterification reaction.

Esters/Time	OH value (mg KOH/g)				Esterification completion, %
	t = 0	t = 1	t = 2	t = f	
SFA-TMP ester	1251	629	415	150	88
SFA-Di-TMP ester	893	450	320	143	84
SFA-PE ester	1642	821	552	247	85
SFA-Di-PE ester	1321	660	442	265	80

Notes: time (t) = 0: Before the reaction, t = 1, 2,..; The hour after the reaction took place; t = f: At the optimal condition.

**Table 9:** The ester yield percentage comparison between two methods of optimization.

Esters	Yield, %	
	Manual*	D-optimal
SFA ester-TMP	87	93
SFA ester Di-TMP	81	89
SFA ester-PE	79	86
SFA ester-Di-PE	74	82

Notes:

* Factor by factor optimization method (manual).

**Table 10:** FTIR signals of SFA PFAD-based ester main functional groups.

Functional group	Wavenumber (cm^−1^)
Stretching vibration of C-H	2922–2849
Stretching vibration of C=O (esters)	1732–1740
Stretching vibration C=O (fatty acid)	1704
scissoring and bending of C-H (methylene)	1460–1466
CH_3_ sym deformation	1380–1390
-C-O-Stretching vibration (ester)	1239–1162
C-H group vibration	721–722

**Table 11:** Lubrication properties of SFA-PFAD-based esters.

Esters	VI	PP (°C)	FP (°C)	OS_T_ (°C)
SFA-TMP	115 ± 3	35 ± 2	270 ± 5	322 ± 2
SFA Di-TMP	125 ± 3	30 ± 2	290 ± 5	260 ± 2
SFA-PE	122 ± 3	33 ± 2	275 ± 5	300 ± 2
SFA-Di-PE	131 ± 3	25 ± 2	310 ± 5	251 ± 2
Dodecanedioate ester (DHD)	181 ± 3	18 ± 2	200 ± 5	201 ± 2

## References

[1] Gryglewicz S Oko F Dicarboxylic acid esters as components of modern synthetic oils Industrial Lubrication and Tribology 2005 57 3 128 132 10.1108/00368790510595101

[2] Knothe G Steidley KR Kinematic viscosity of biodiesel fuel components and related compounds. Influence of compound structure and comparison to petrodiesel fuel components Fuel 2005 84 9 1059 1065 10.1016/j.fuel.2005.01.016

[3] Erhan SZ Industrial uses of plant oils USA Boca Raton, AOCS Press 2005

[4] Nagendramma P Kaul S Development of ecofriendly/biodegradable lubricants: An overview Renewable and Sustainable Energy Reviews 2012 16 1 764 774 10.1016/j.rser.2011.09.002

[5] Boyde S Hydrolytic stability of synthetic ester lubricants Journal of Synthetic Lubrication 2000 16 4 297 312 10.1002/jsl.3000160403

[6] Kiss AA Bildea CS A review of biodiesel production by integrated reactive separation technologies Journal of Chemical Technology & Biotechnology 2012 87 7 861 879 10.1002/jctb.3785

[7] Hama S Kondo A Enzymatic biodiesel production: an overview of potential feedstocks and process development Bioresource Technology 2013 135 386 395 10.1016/j.biortech.2012.08.014 22985827

[8] Calero J Luna D Sancho ED Luna C Bautista FM An overview on glycerol-free processes for the production of renewable liquid biofuels, applicable in diesel engines Renewable and Sustainable Energy Reviews 2015 42 1437 1452 10.1016/j.rser.2014.11.007

[9] Vernon FMJ Standard Brands (UK) Ltd Firelighter with palm fatty acid distillate U.S. Patent Application 15/749,703 2018

[10] Kapor NZA Maniam GP Rahim MHA Yusoff MM Palm fatty acid distillate as a potential source for biodiesel production - a review Journal of Cleaner Production 2017 143 1 9 10.1016/j.jclepro.2016.12.163

[11] Ping BTY Yusof M Characteristics and properties of fatty acid distillates from palm oil Oil Palm Bulletin 2009 59 5 11

[12] Majd AJ Mohamad Firdaus MY Salimon J Murad B Separation of saturated and unsaturated fatty acids of palm fatty acid distilled via low-temperature methanol crystallization Malaysian Journal of Chemistry 2019 21 8 16

[13] Baharudin KB Taufiq-Yap YH Hunns J Isaacs M Wilson K Mesoporous NiO/Al-SBA-15 catalysts for solvent-free deoxygenation of palm fatty acid distillate Microporous and Mesoporous Materials 2019 276 13 22 10.1016/j.micromeso.2018.09.014

[14] Widodo S Khoiruddin K Ariono D Subagjo S Wenten IG Re-refining of waste engine oil using ultrafiltration membrane Journal of Environmental Chemical Engineering 2020 8 3 103789 10.1016/j.jece.2020.103789

[15] Koh MY Tinia TI Idris A Synthesis of palm based biolubricant in an oscillatory flow reactor (OFR) Industrial Crops and Products 2014 52 567 574 10.1016/j.indcrop.2013.10.042

[16] Nowicki J Stańczyk D Drabik J Mosio-Mosiewski J Woszczyński P Synthesis of fatty acid esters of selected higher polyols over homogeneous metallic catalysts Journal of the American Oil Chemists’ Society 2016 93 7 973 981 10.1007/s11746-016-2840-7 27418694 PMC4919386

[17] Japir AA Salih N Salimon J Synthesis and characterization of biodegradable palm palmitic acid based bioplastic Turkish Journal of Chemistry 2021 45 585 599 10.3906/kim-2011-31 34385854 PMC8326477

[18] Bahadi M Salih N Salimon J D-Optimal design optimization for the separation of oleic acid from Malaysian high free fatty acid crude palm oil fatty acids mixture using urea complex fractionation Applied Science and Engineering Progress 2021 14 2 175 186 10.14416/j.asep.2021.03.004

[19] Stachowiak GW Batchelor AW Engineering tribology USA New York, Elsevier Butterworth-Heinemann 2005

[20] Almasvandi MH Rahimi M Tagheie Y Microfluidic cold stripping of H_2_S from crude oil in low temperature and natural gas consumption Journal of Natural Gas Science and Engineering 2016 34 499 508 10.1016/j.jngse.2016.07.021

[21] Zhang Z Zheng H Optimization for decolorization of azo dye acid green 20 by ultrasound and H_2_O_2_ using response surface methodology Journal of Hazardous Materials 2009 172 2–3 1388 1393 10.1016/j.jhazmat.2009.07.146 19717231

[22] Eriksson L Jaworska J Worth AP Cronin MT McDowell RM Methods for reliability and uncertainty assessment and for applicability evaluations of classification-and regression-based QSARs Environmental Health Perspectives 2003 111 10 1361 1375 10.1289/ehp.5758 12896860 PMC1241620

[23] Myers RH Montgomery DC Vining GG Borror CM Kowalski SM Response surface methodology: a retrospective and literature survey Journal of Quality Technology 2004 36 1 53 77 10.1080/00224065.2004.11980252

[24] Awang R Ghazuli MR Basri M Immobilization of lipase from Candida Rugosa on palm-based polyurethane foam as a support material American Journal of Biochemistry and Biotechnology 2007 3 3 163 166 10.3844/ajbbsp.2007.163.166

[25] Sharma BK Adhvaryu A Liu Z Erhan SZ Chemical modification of vegetable oils for lubricant applications Journal of the American Oil Chemists’ Society 2006 83 2 129 136 10.1007/s11746-006-1185-z

[26] Bahadi M Salih N Salimon J Synthesis and characterization of green biodegradable palm oleic acid based polyester Biointerface Research in Applied Chemistry 2021 11 6 14359 14371 10.33263/BRIAC116.1435914371

[27] Pavia DL Lampman GM Kriz GS Vyvyan JR Introduction to spectroscopy 5th Ed Cengage Learning, Inc USA 2015

[28] Alexandri E Ahmed R Siddiqui H Choudhary MI Tsiafoulis CG Gerothanassis IP High resolution NMR spectroscopy as a structural and analytical tool for unsaturated lipids in solution Molecules 2017 22 10 1663 10.3390/molecules22101663 28981459 PMC6151582

[29] Mortier RM Fox MF Orszulik S Chemistry and technology of lubricants 3rd ed New York Springer 2010

[30] Lye YN Salih N Salimon J Optimization of partial epoxidation on *Jatropha curcas* oil based methyl linoleate using urea-hydrogen peroxide and methyltrioxorhenium catalyst Applied Science and Engineering Progress 2021 14 1 89 99 10.14416/j.asep.2020.12.006

[31] Salih N Salimon J A Review on eco-friendly green biolubricants from renewable and sustainable plant oil sources Biointerface Research in Applied Chemistry 2021 11 5 13303 13327 10.33263/BRIAC115.1330313327

[32] Nor NM Salih N Salimon J Chemically modified *Jatropha curcas* oil for biolubricant applications Hemijska Industrija 2021 75 2 117 128 10.2298/hemind200809009n

[33] Salimon J Salih N Modification of epoxidized ricinoleic acid for biolubricant base oil with improved flash and pour points Asian Journal of Chemistry 2010 22 5468 5476

[34] Samidin S Salih N Salimon J Synthesis and characterization of trimethylolpropane based esters as green biolubricant basestock Biointerface Research in Applied Chemistry 2021 11 5 13638 13651 10.33263/BRIAC115.1363813651

[35] Polansky R Prosr P Vik R Moravcova D Pihera J Comparison of the mineral oil lifetime estimates obtained by differential scanning calorimetry, infrared spectroscopy, and dielectric dissipation factor measurements Thermochimica Acta 2017 647 86 93 10.1016/j.tca.2016.12.002

[36] Nor NM Salih N Salimon J Optimization of the ring opening of epoxidized palm oil using D-optimal design Asian Journal of Chemistry 2021 33 1 67 75 10.14233/ajchem.2021.22938

[37] Salih N Salimon J A Review on new trends, challenges and prospects of ecofriendly friendly green food-grade biolubricants Biointerface Research in Applied Chemistry 2022 12 1 1185 1207 10.33263/BRIAC121.11851207

[38] Arbain NH Salimon J The effects of various acid catalyst on the esterification of *Jatropha curcas* oil based trimethylolpropane ester as biolubricant base stock E-Journal of Chemistry 2011 8 1 33 40 10.1155/2011/789374

